# Conserved and Divergent Roles of Bcr1 and CFEM Proteins in *Candida parapsilosis* and *Candida albicans*

**DOI:** 10.1371/journal.pone.0028151

**Published:** 2011-12-01

**Authors:** Chen Ding, Genevieve M. Vidanes, Sarah L. Maguire, Alessandro Guida, John M. Synnott, David R. Andes, Geraldine Butler

**Affiliations:** 1 School of Biomolecular and Biomedical Science, Conway Institute, University College Dublin, Belfield, Dublin, Ireland; 2 School of Medicine and Medical Science, Conway Institute, University College Dublin, Belfield, Dublin, Ireland; 3 Department of Microbiology and Immunology, University of Wisconsin, Madison, Wisconsin, United States of America; Institute of Developmental Biology and Cancer Research, France

## Abstract

*Candida parapsilosis* is a pathogenic fungus that is major cause of hospital-acquired infection, predominantly due to growth as biofilms on indwelling medical devices. It is related to *Candida albicans*, which remains the most common cause of candidiasis disease in humans. The transcription factor Bcr1 is an important regulator of biofilm formation *in vitro* in both *C. parapsilosis* and *C. albicans*. We show here that *C. parapsilosis* Bcr1 is required for *in vivo* biofilm development in a rat catheter model, like *C. albicans*. By comparing the transcription profiles of a *bcr1* deletion in both species we found that regulation of expression of the CFEM family is conserved. In *C. albicans*, three of the five CFEM cell wall proteins (Rbt5, Pga7 and Csa1) are associated with both biofilm formation and acquisition of iron from heme, which is an important virulence characteristic. In *C. parapsilosis*, the CFEM family has undergone an expansion to 7 members. Expression of three genes (*CFEM2*, *CFEM3*, and *CFEM6*) is dependent on Bcr1, and is induced in low iron conditions. All three are involved in the acquisition of iron from heme. However, deletion of the three CFEM genes has no effect on biofilm formation in *C. parapsilosis*. Our data suggest that the role of the CFEM family in iron acquisition is conserved between *C. albicans* and *C. parapsilosis*, but their role in biofilm formation is not.

## Introduction

*Candida* species are among the most common causes of nosocomial bloodstream infection, and have associated mortality rates ranging from 28–59% [1], [2]. *Candida albicans* is still the most commonly isolated, but other *Candida* species such as *C. glabrata*, *C. parapsilosis* and *C. krusei* are increasingly reported [1], [2], [3]. *C. parapsilosis* in particular is found on the hands of health care workers, and has been responsible for several outbreaks of infection [4], [5], [6], [7], [8].

Although often found as commensal organisms with humans, *Candida* species are also capable of growth as antifungal-resistant biofilms on non-biological surfaces such as medical equipment. Surgical intervention and the increasingly invasive nature of medical care, supported by the use of catheters or intravenous devices, provide opportunities for the dissemination of these biofilm-forming fungi [9]. Whereas all *Candida* species form biofilms on solid surfaces, the structures are highly variable [10], [11]. In *C. albicans*, biofilms are multilayered and contain yeast cells, pseudohyphae and hyphae [12]. Biofilm development by *C. albicans* has been well characterized, and occurs in several stages (reviewed in [10], [13]). Adherence of yeast cells to the substrate is followed by an intermediate stage where hyphae are formed and an extracellular matrix is generated. A mature biofilm consists of densely packed hyphae and yeast cells surrounded by the extracellular matrix, consisting mostly of polysaccharides [14]. *C. parapsilosis* biofilms in contrast consist of a dense network of yeast cells and pseudohyphae, but they also contain large amounts of carbohydrate [11], [15].

*BCR1* (Biofilm and Cell wall Regulator 1) is a conserved fungal transcription factor required for biofilm formation in both *C. albicans* and *C. parapsilosis* [16], [17], [18]. Some major targets of Bcr1 in *C. albicans* include genes that encode for adhesins and cell-wall proteins (*ALS1*, *ALS3*, *HWP1*, and *RBT5* and related genes), suggesting that Bcr1 is involved in the early adhesion stage of biofilm development [17], [18], [19], [20]. Although the *C. parapsilosis* genome contains members of all these gene families, there are substantial differences between the species [21]. For example, *ALS3*, a major adhesin, is found only in *C. albicans*, and not in other *Candida* species [22]. Rbt5 is a member of the CFEM (common in fungal extracellular membranes) family of proteins with an eight-cysteine domain resembling an EGF domain, which was originally identified in *Magnaporthe grisea* [23], [24]. Many family members contain putative GPI-anchors, and several are identified with pathogenesis. EGF-like domains are often found in the extracellular regions of membrane proteins, and Kulkarni et al [23] suggested that CFEM proteins may act as cell surface receptors or as adhesins. There are five members of the CFEM family in *C. albicans*, of which at least three (*RBT5*, *PGA10* and *CSA1*) are important for biofilm development [25]. In *C. parapsilosis* the family has undergone an expansion to seven members, which includes tandem duplicates of orthologs of *C. albicans RBT5*, *PGA10* and *CSA1*.

The ability to acquire essential iron from host proteins is critical for survival of pathogenic fungi. Iron is generally a limiting nutrient, and is often sequestered by the host [26]. *C. albicans* has multiple mechanisms for utilizing iron sources from the environment, including a reductive pathway and transport of heterologous siderophores (reviewed in [27]). Some Bcr1 targets in *C. albicans* also play a role in acquiring iron from host proteins. These include two CFEM proteins, Rbt5 and Pga10, which act as receptors for hemoglobin, allowing endocytosis of the host iron complex [28], [29]. Als3, uniquely among the ALS family of adhesins, binds to ferritin, enabling its use as a source of iron [30].

We describe here an analysis of the role of Bcr1 in *C. parapsilosis*. We show for the first time that *C. parapsilosis* generates biofilms *in vivo* in a rat catheter model, and that *BCR1* is required for this process. Whereas there is little overlap among the targets of Bcr1 in the two species, regulation of the CFEM family is conserved. Moreover, the role of CFEM proteins in iron acquisition is conserved. However, unlike *C. albicans*, the CFEM genes are not required for biofilm formation in *C. parapsilosis*.

## Results

### *BCR1* is required for *in vivo* biofilm formation in *C. parapsilosis*

**Figure 1:**
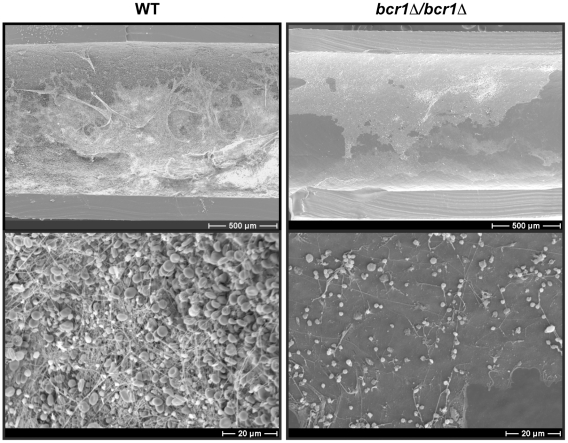
*BCR1* is required for *C. parapsilosis* biofilm formation *in vivo*. Central venous catheters were introduced into rats and inoculated with *C. parapsilosis* wildtype (CLIB214) or *bcr1* deletion (CDb71) strains. Following initial adhesion, the cells were flushed and locked with heparinized 0.85% NaCl. The catheters were removed after 24 h and visualized at two magnifications by SEM.

To date, most investigations of biofilm development by *C. parapsilosis* have used *in vitro* systems, such as growth in 96-well plates or on silicon squares [16], [31], [32], [33], [34]. However, in *C. albicans*, mutants do not always behave the same in *in vitro* and *in vivo* models. For example, deleting *ALS3* has a dramatic effect on biofilm development *in vitro*, but not *in vivo* [17]. We therefore tested the ability of *C. parapsilosis* to grow as biofilms in the rat catheter model, designed for investigating *C. albicans* biofilm development [35]. Figure 1 shows that *C. parapsilosis* wildtype cells produce a robust biofilm 24 h after the introduction of cells into the catheter. Although the structure differs from *C. albicans* biofilms in that there are no hyphae present, a thick biofilm layer is formed. In contrast, strains carrying a deletion of *BCR1* [16] form a very thin and sparse layer of cells (Figure 1), showing that *BCR1* is also required for biofilm formation *in vivo* as well as *in vitro* [16]. These experiments illustrate the robustness of the rat catheter biofilm model, and demonstrate that it can be extended to species that do not generate hyphae.

### Identification of targets of Bcr1 in *C. parapsilosis*

We previously described the construction of a *bcr1* knockout in *C. parapsilosis* using a nourseothricin-resistant *SAT1*-flipper cassette, which can be recycled and reused to disrupt multiple alleles [16]. However, the method is relatively slow and reintroducing the *BCR1* gene did not fully reconstitute the phenotype [16]. To facilitate the identification of targets, we generated a second *bcr1* deletion using a different method. Firstly, the *HIS1* gene was deleted in a *ura3* auxotrophic background [16] using the *SAT1*-flipper cassette (Figure S1A). The two *BCR1* alleles were then disrupted in this *ura3Δ his1Δ* background by replacing one allele with *URA3* and the other with *HIS1* (Figure S1B). The *bcr1Δ::FRT/bcr1Δ::FRT* strain (CDb71) described previously [16] and the *bcr1Δ::URA3/bcr1Δ::HIS1* strain (CDUHB6) were grown in biofilm inducing conditions (SD, 50 mM glucose, and 10% FBS at 37°C), and used for expression profiling as described in Rossignol et al [36]. The data from both knockouts were considered together to remove any artifacts associated with the individual knockouts, such as strain-specific effects that are unrelated to Bcr1. We also determined the transcriptional profile of *C. albicans BCR1/BCR1* and *bcr1Δ*/*bcr1Δ* strains (DAY286 and CJN702, respectively, gifts from A. Mitchell) grown in the same conditions, to facilitate a comparison of the two species. We included the data from transcriptional profiling of the *C. albicans bcr1* deletion strain grown in Spider media previously reported by Nobile and Mitchell [18].

**Figure 2:**
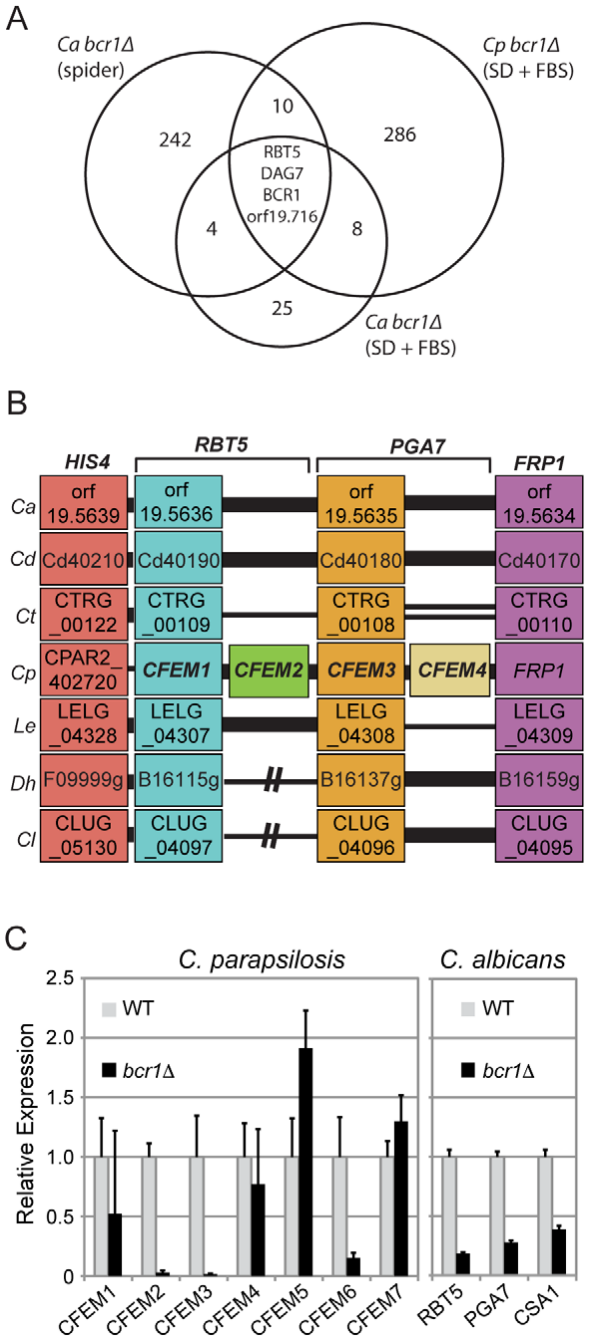
Bcr1 regulates expression of CFEM genes in *C. parapsilosis* and *C. albicans*. A. Intersection between the targets of Bcr1 in *C. albicans* and *C. parapsilosis*. The data from *C. albicans* cells grown in Spider media is taken from Nobile and Mitchell [18]. The lists of genes regulated in both species grown in SD+FBS media is provided in Table S1 and Table S2. B. Gene order around the CFEM genes in seven yeast species (adapted from the Candida Gene Order Browser, [69]). The order of *HIS4*, *RBT5*, *PGA7* and *FRP1* is highly conserved across most *Candida* species. In *C. parapsilosis*, however, *RBT5* and *PGA7* have undergone gene duplication, resulting in four adjacent genes, named *CFEM1*, *CFEM2*, *CFEM3* and *CFEM4*. *CFEM5* and *CFEM6* are also adjacent to each other elsewhere in the genome (not shown). Thick black lines represent adjacent genes. Two thin black lines represent a gap of less than 5 genes, and one thin line represents a gap of less than 20 genes. A black line with breaks indicates genes that are not on the same chromosomes. Ca: *C. albicans*; Cd: *Candida dubliniensis*; Ct: *Candida tropicalis*; Cp: *C. parapsilosis*; Le: *Lodderomyces elongisporus*; Dh: *Debaryomyces hansenii*; Cl: *Candida lusitaniae*. C. Expression of CFEM members (*CFEM1* to *CFEM7*) in *C. parapsilosis* and the homologous genes in *C. albicans* was determined using qRT-PCR. All strains were grown in SD medium supplemented with 50 mM glucose and 10% FBS for 5 h at 37°C.

Somewhat surprisingly, there is very little overlap between the targets of Bcr1 in *C. albicans* and *C. parapsilosis* (Figure 2A). Only four genes are present in the intersection of the three data sets, and one is *BCR1*, which is deleted in all strains. However, one notable observation is that *RBT5*, a member of the CFEM family, is also present in the intersection of the three data sets. Of the remaining two genes in the intersection, one (*orf19.716*) is differently regulated in *C. albicans* and *C. parapsilosis*, and the other (*DAG7*) has increased expression in both species. Expression of these genes was not investigated further.

There are five members of the CFEM family in *C. albicans* (*PGA7*, *PGA10*, *RBT5*, *CSA1* and *CSA2*) and seven members in *C. parapsilosis*, which we have named *CFEM1*-*CFEM7*. Four of these (*CFEM1*-*4*) are tandemly arranged, and are syntenic with *RBT5* and *PGA7* (Figure 2B). Other *Candida* clade species contain only two genes in this region. Examination of synteny, together with phylogenetic analysis, suggests that both *RBT5* and *PGA7* have undergone single gene duplications in *C. parapsilosis*, leading to the formation of *CFEM1/CFEM2* and *CFEM3/CFEM4*, respectively. Similarly, *CFEM5* and *CFEM6* are orthologous with *CSA1* (not shown). However, *CFEM7* has no observable ortholog within the *Candida* clade (not shown) and may represent a relatively recent evolutionary addition to the CFEM family specific to *C. parapsilosis*.

Because the CFEM genes are not directly orthologous in the two species, we used qRT-PCR to determine the role of Bcr1 in regulating expression of most of the related family members in both (Figure 2C). Firstly, we showed that expression of three family members (*RBT5*, *PGA7* and *CSA1*) is reduced in *C. albicans bcr1Δ*, which confirms and extends some previously published observations [18]. One member of each orthologous pair in *C. parapsilosis*, *CFEM2*, *CFEM3* and *CFEM6*, is downregulated in the *bcr1Δ* mutant (Figure 2C and Table S1). In contrast, expression of *CFEM1*, *CFEM4*, *CFEM5*, and *CFEM7* is essentially unchanged. Thus, the regulation of some CFEM genes by the biofilm transcription factor Bcr1 is conserved between *C. albicans* and *C. parapsilosis*, but not all members of the family are regulated by Bcr1 in *C. parapsilosis*.

### Deletions of CFEM genes do not affect biofilm formation in *C. parapsilosis*

**Figure 3:**
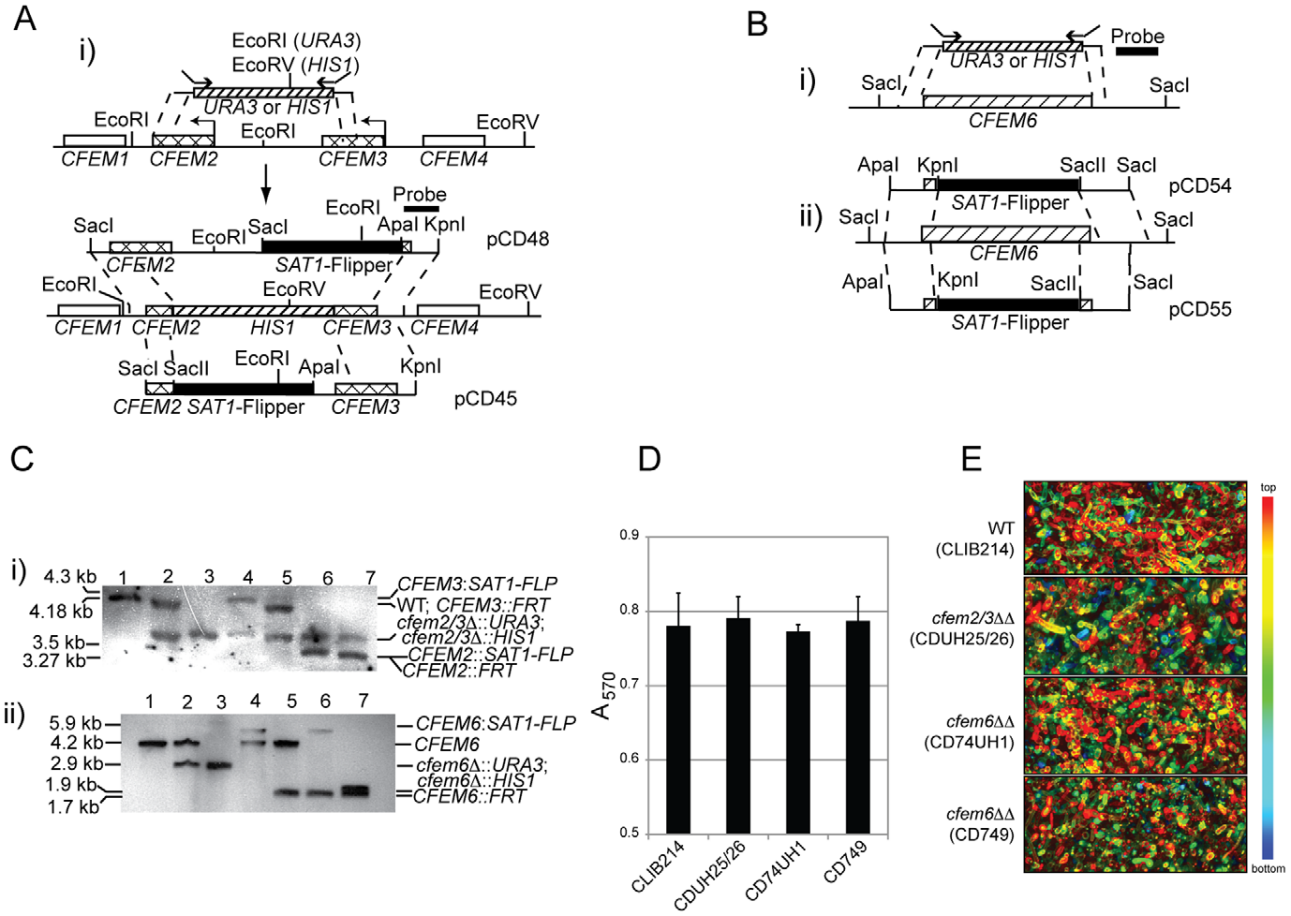
*C. parapsilosis* CFEM genes are not required for biofilm formation. A. *C. parapsilosis CFEM2* and *CFEM3* were deleted simultaneously by replacement with *CaURA3* and *CaHIS1* to generate a homozygous *cfem2Δ/cfem3Δ* strain (CDUH25/26). Complemented strains contain either *CFEM2* or *CFEM3*, re-introduced by using the *SAT1*-flipper cassette at the *cfem2Δ/cfem3Δ::HIS1* locus. B. The homozygous *cfem6Δ* strains were generated by two methods. (i) Strain CD74UH1 was made by replacing each *CFEM6* allele with *CaURA3* and *CaHIS1*. (ii) Strain CD749 was created by two rounds of *CFEM6* gene deletion with the recyclable *SAT1*-flipper. C. (i) The construction of the *cfem2Δ/cfem3Δ* homozygous (CDUH25/26) and *CFEM2* and *CFEM3* complemented strains (CD252 and CDC262, respectively) was confirmed by Southern blot using a probe hybridizing to promoter sequence from *CFEM3*. The expected sizes are described in Materials and Methods. Lane 1: CLIB214 (*C. parapsilosis* wildtype strain); lane 2: CDUH2526his (*CFEM2+3*/*cfem2+3Δ::HIS1*); lane 3: CDUH25/26 (*cfem2+3Δ::URA3/cfem2+3Δ::HIS1*); lane 4: CD26 (*cfem2Δ+CFEM3::SAT1-FLP/cfem2+3Δ::URA3*); lane 5: CD262 (*cfem2Δ+CFEM3::FRT/cfem2+3Δ::URA3*); lane 6: CD25 (*CFEM2Δ+cfem3::SAT1-FLP/cfem2+cfem3Δ::URA3*); lane 7: CD254 (*CFEM2+cfem3Δ::FRT/cfem2+3Δ::URA3*). (ii) The construction of *CFEM6* was confirmed by Southern blot using a probe hybridizing to sequence from the 3′ end of *CFEM6*. Lane 1: CLIB214 (*C. parapsilosis* wildtype strain); lane 2: CD74U2 (*CFEM6/cfem6Δ::URA3*); lane 3:CD74UH1 (*cfem6Δ::HIS1/cfem6Δ::URA3*); lane 4: CD741 (*cfem6Δ::SAT1-FLP/CFEM6*); lane 5: CD745 (*cfem6Δ::FRT/CFEM6*); lane 6: CD746 (*cfem6Δ::FRT/cfem6Δ::SAT1-FLP*); lane 7:CD749 (*cfem6Δ::FRT/cfem6Δ::FRT*). D. Biofilms formed by *C. parapsilosis* CLIB214 (wildtype), CDUH25/26 (*cfem2Δ/cfem3Δ*), CD74UH1 (*cfem6Δ::URA3/cfem6Δ::HIS1*), and CD749 (*cfem6Δ::FRT/cfem6Δ::FRT*) were measured in 96-well plates as previously described [31]. Biofilms were stained using crystal violent and the A570 was measured. Three biological replicates used, each replicated eight times on the same plate. E. Biofilms grown on silicon squares by *C. parapsilosis* were visualized using confocal microscopy as previously described [16]. The structure of biofilm matrix was obtained using a 40× lens, and the depth of biofilm was measured using a 10× lens. The depth of the biofilm in *C. parapsilosis* strains ranges from 90 to 120 µm. The depths for the individual strains are approximately 116 µm in CLIB214; 120 µm in CDUh25/26; 98 µm in CD74UH1; and 96 µm in CD749.

Deletion of *BCR1* in either *C. albicans* or *C. parapsilosis* results in an inability to form biofilms (Figure 1, [16], [18]. In addition, deleting *RBT5*, *PGA10* or *CSA1* in *C. albicans* also reduces biofilm development [25]. This suggests that the role of Bcr1 in regulating biofilm development is partially effected through controlling expression of the CFEM family. We therefore tested the role of the Bcr1-regulated members of the CFEM family in *C. parapsilosis* on biofilm development. *CFEM2* and *CFEM3* were deleted simultaneously by replacement with *URA3* and *HIS1* as they are adjacent in the genome, and the wildtype genes were subsequently individually re-introduced using the *SAT1* flipper cassette. *CFEM6* was deleted separately (Figure 3).

We determined the ability of the *cfem2Δ/cfem3Δ* and *cfem6Δ* strains to form biofilms on microtiter plates and on silicone squares. Surprisingly, neither deletion had a measurable effect on biofilm mass or structure, as ascertained by crystal violet staining of the microtiter plates (Figure 3D) and confocal microscopy of the silicone squares (Figure 3E). These results suggest that neither *CFEM2*, *CFEM3* nor *CFEM6* are required for biofilm growth in *C. parapsilosis*. However, the possibility remains that other CFEM genes may compensate for this function in the *cfem2Δ/cfem3Δ* and *cfem6Δ* mutants.

### Role of the CFEM family in iron acquisition in *C. parapsilosis*

**Figure 4:**
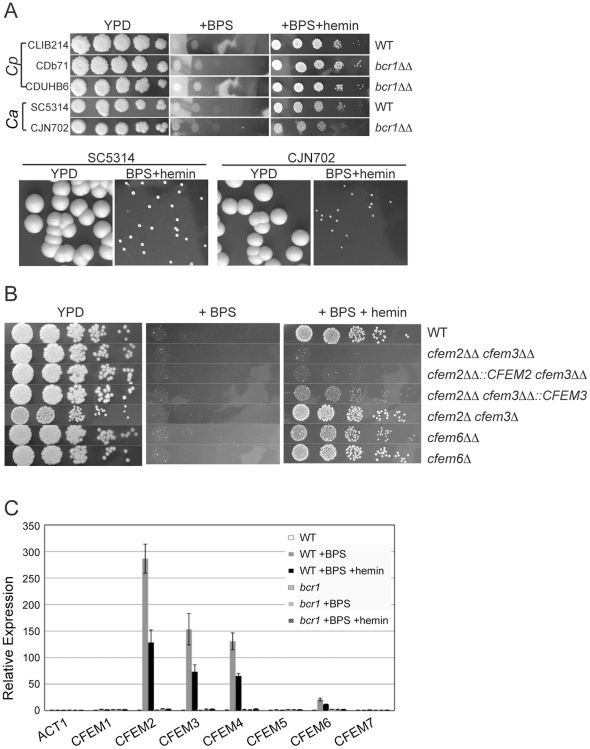
*CFEM2, CFEM3*, and *CFEM6* are required for heme utilization. A. *C. parapsilosis strains* CLIB214 (wild-type), CDb71 (*bcr1Δ::FRT/bcr1Δ::FRT*) and CDUHB6 (*bcr1Δ::HIS1/bcr1Δ::URA3*) and *C. albicans* strains SC5314 (wild-type) and CJN702 (*bcr1Δ*) were serially diluted on YPD plates, YPD supplemented with 1 mM BPS, and YPD supplemented with 1 mM BPS and 2 µM hemin for 7 days at 30°C. Deleting *BCR1* in *C. albicans* reduces colony size, which is shown by photographing individual colonies on YPD plates supplemented with 1 mM BPS and 2 µM hemin after 7 days. Pictures were taken on the same day and magnification. B. *C. parapsilosis* strains containing deletions of CFEM genes were serially diluted on plates as described in (A) and were incubated at 30°C for two days (YPD plates) or 14 days (BPS+/−hemin) before photographing. Strains shown in the following order: CLIB214, CDUH25/26, CDUH254, CDUH262, CDUH25/26 his, CD748, CD743. C. Expression of *C. parapsilosis* CFEM genes was determined using qRT-PCR. RNA was extracted from CLIB214 and CDb71 cells grown in SD+50 mM glucose with no BPS, 200 µM BPS, and 200 µM BPS supplemented with 2 µM hemin for 5 h at 37°C (p values:*<0.05).

Many species, including *C. albicans* and *S. cerevisiae*, induce multiple pathways for iron acquisition during growth in iron-depleted media [27], [37]. In *C. albicans*, the CFEM proteins Rbt5 and Pga10 are required specifically for heme-iron utilization [28], [29]. We first tested if Bcr1, as a regulator of CFEM expression, is also involved in iron acquisition. We plated strains on rich, iron-depleted (BPS), or hemin-supplemented BPS plates. Both *C. albicans* and *C. parapsilosis* grow very poorly under iron-depleted conditions (Figure 4A). Growth of both species is rescued by the addition of hemin, although *C. parapsilosis* is better able to utilize hemin as a sole iron source than *C. albicans*. *C. albicans bcr1Δ* colonies are smaller than wildtype colonies grown for an equivalent time on hemin plates (Figure 4A), suggesting that Bcr1 may contribute to the regulation of heme utilization in this species. In contrast, the absence of *BCR1* has no obvious effect on heme utilization in *C. parapsilosis* (Figure 4A).

To test if the CFEM genes in *C parapsilosis* play any role in acquisition of iron from heme, we plated dilutions of *cfem2Δ/cfem3Δ* and *cfem6Δ* strains on iron-depleted (BPS) or hemin-supplemented (BPS+hemin) plates (Figure 4B). The homozygous *cfem2Δ/cfem3Δ* strain can no longer grow on plates containing hemin as a sole source of iron. Reintroducing a single copy of *CFEM2* alone does not significantly restore growth, but reintroducing *CFEM3* partially restores growth (Figure 4B). However, it appears that both *CFEM2* and *CFEM3* (or perhaps two alleles of either) are required to obtain wildtype levels of growth (compare the reconstituted strains with the heterozygote). Deleting *CFEM6* also reduces growth on hemin, although the effect is not as dramatic as in the *cfem2Δ/cfem3Δ* homozygous knockout (Figure 4B). Thus, *CFEM6* is partially required while *CFEM2* and *CFEM3* are vital for heme utilization in *C. parapsilosis*.

### Iron-depletion induces CFEM gene expression in *C. parapsilosis*

We used qRT-PCR to further examine the effect of iron depletion on expression of the CFEM family in *C. parapsilosis*. Cells were grown in the presence of the iron chelator BPS, and in the absence of serum (FBS) which we induces expression of the CFEM family (not shown). Expression of *CFEM2*, *CFEM3*, *CFEM4* and *CFEM6* is induced when iron levels are low, and expression is reduced when hemin is added (Figure 4C). Induction of expression in low iron requires Bcr1. Expression of *CFEM1*, *CFEM5* and *CFEM7* is not reproducibly induced by iron depletion (Figure 4C) and is not regulated by Bcr1 (Figure 2C). The expression data suggests that *CFEM4* may also play a role in iron acquisition from heme, which we have not tested.

To determine how important the CFEM family is for iron acquisition in *C. parapsilosis*, we determined the global transcriptional profile of cells grown in iron-depleted conditions. We identified 59 genes with increased expression, and 89 genes with decreased expression (Table S3). As expected, we observed significant increases in expression of genes associated with cellular iron ion homeostasis and iron ion transport, such as *FTH1*, *FRE9*, and *FRE10* (Table 1, Table S3). In contrast, expression of heme-containing and iron-sulfur proteins (e.g. *YHB1*, *SDH2*, *ISA1*) and of all mitochondrial genes is reduced (Table S3). Overall the response of *C. parapsilosis* and *C. albicans* to low iron conditions is very similar [38], [39]. Three of the CFEM family (*CFEM2*, *CFEM3* and *CFEM6*) are among the genes with the highest increases in expression (logFC>1.9), confirming that they play an important role in the iron response (Table 1).

**Table 1:** Selected *C. parapsilosis* genes differentially expressed in low iron conditions.

*C. parapsilosis gene name* 1	*C. parapsilosis ID* 2	*C. albicans* homolog	Log FC
**CFEM family**			
*CFEM2*	*CPAR2_402910*	*RBT5*	3.49
*CFEM3*	*CPAR2_402900*	*PGA10*	2.92
*CFEM6*	*CPAR2_300120*	*CSA1*	1.96
**Reductive iron transport**			
*CPAG_00389*	*CPAR2_300630*	*orf19.7077 (FRE7-like)*	4.92
*CPAG_00390*	*CPAR2_300620*	*orf19.7077 (FRE7-like)*	3.68
*CPAG_00393*	*CPAR2_300580*	*orf19.7077 (FRE7-like)*	3.65
*CPAG_03224*	*CPAR2_805630*	*FTR1-like*	3.11
*CPAG_03730*	*CPAR2_603590*	*FET99*	2.62
*CPAG_03065*	*CPAR2_210140*	*FRE9*	2.35
*CPAG_01229*	*CPAR2_210100*	*FTH1*	2.55
*CPAG_03792*	*CPAR2_602990*	*CTR1*	2.25
*CPAG_04019*	*CPAR2_401740*	*FRE10*	0.96
*CPAG_03370*	*CPAR2_303120*	*CCC2*	0.91
*CPAG_00623*	*CPAR2_300130*	*FRP2*	0.69
**Other iron transporters**			
*CPAG_03610*	*CPAR2_105690*	*HMX1*	0.81
**Transcription factors/regulatory proteins**			
*CPAG_02488*	*CPAR2_407950*	*CTH1 (orf19.5334)*	1.61
*CPAG_04485*	*CPAR2_801430*	*SEF1*	0.94
*CPAG_01899*	*CPAR2_209090*	*HAP43*	0.84
*CPAG_03938*	*CPAR2_301500*	*MAC1*	0.75
*CPAG_04289*	*CPAR2_700810*	*SFU1*	−1.09

1 *C. parapsilosis* ID from http://www.broadinstitute.org/annotation/genome/candida_group/MultiHome.html.

2 *C. parapsilosis* ID from accession no. HE605202-HE605210.

## Discussion

Both biofilm formation and iron acquisition are contributing factors to the pathogenicity of *Candida* species. Our analysis shows that the Bcr1 transcription factor is an important regulator of biofilm development *in vitro* and *in vivo* in the two species, and that regulation of expression of some members of the CFEM family is conserved [16], [17], [18]. However, the Bcr1-dependent CFEM genes do not play a role in biofilm development in *C. parapsilosis*.

Transcriptional profiling reveals that *RBT5/CFEM2* is regulated by Bcr1 in both *C. albicans* and *C. parapsilosis*, and follow-up analysis confirmed that several members of the CFEM family are regulated in the two species. There is very little other overlap between the targets of Bcr1 (Figure 2C, Tables S1 and S2). Both species were grown in conditions that promote biofilm production in *C. parapsilosis* (SD+10% FBS at 37°C) [16]. However, these conditions are also ideal for hyphal growth by *C. albicans*. We included published data from transcriptional profiling of a *C. albicans bcr1* deletion grown in Spider media, which also induces hyphal growth [18]. Several genes associated with both biofilm development and with hyphal growth (including members of the CFEM family) are differentially expressed in the two experiments. Four genes have altered expression in *C. albicans bcr1* irrespective of growth conditions (*ALS3*, *ECE1*, *PTP3* and *CFL2*). None of these have direct orthologs in *C. parapsilosis*, although apart from *PTP3*, they are all members of gene families that are represented in the two species. The first three genes are induced in hyphae in *C. albicans* [40], [41], [42]. It is therefore likely that in *C. albicans*, Bcr1 plays a role in regulating expression of hyphal-induced genes that is not conserved in *C. parapsilosis*.

There are ten genes that are differentially expressed in both *C. albicans* and *C. parapsilosis* grown in the same conditions (SD+10% FBS). However, many of these have reduced expression in *C. albicans* and increased expression in *C. parapsilosis* (Table S4), and are therefore unlikely to form part of the conserved Bcr1 regulon.

In *C. albicans*, expression of the CFEM genes *RBT5*, *PGA7*, and to a lesser extent *CSA1*, is dependent on Bcr1 (Fig. 2C). In *C. parapsilosis*, each of these genes has been duplicated, generating *CFEM1* to *CFEM6*; *CFEM1* to *CFEM4* are found in tandem, and *CFEM5* and *CFEM6* are also adjacent but a different location to the other four. The final member of the *C. parapsilosis* family, *CFEM7*, does not have a syntenic ortholog in *C. albicans*. Within the three gene pairs, expression of one (*CFEM2*, *CFEM3* and *CFEM6*) is highly dependent on Bcr1, whereas expression of the other member of the pair is reduced only slightly, if at all, in a *bcr1* deletion. In fact, expression of *CFEM5* may be repressed (Fig. 2C). This suggests that following the gene duplication event(s), one copy of each pair retained the Bcr1-dependent regulation, which was lost in the second copy. It is not yet clear what the biological significance of the gene duplication events is or why the regulation by Bcr1 is different.

CFEM genes in *C. albicans* play an important role in the acquisition of iron from host proteins [28], [29]. Expression of *RBT5* is highly induced under low iron conditions [28], [38], [39] and the protein binds heme and is required for endocytosis of hemoglobin [28], [29]. Deleting *PGA10* (also known as *RBT51*) has no obvious affect on growth on heme, but when introduced into *S. cerevisiae* it confers on this species the ability to use heme as an iron source [28]. *CSA1* is also required for maximal binding of heme [28]. We used RT-PCR to test the response of the entire CFEM family in *C. parapsilosis* to low iron conditions, and showed that expression of *CFEM2*, *CFEM3*, *CFEM4*, and *CFEM6* are greatly induced. The iron-dependent response was partially alleviated by adding back hemin. Bcr1 regulates these genes, apart from CFEM4, in the presence of serum (Figure 2C) and induction of expression of all four in iron-depleted conditions is dependent on Bcr1 (Figure 4). We therefore tested the role of Bcr1 in iron acquisition. However, deleting *bcr1* has no obvious effect on cell growth on hemin as a sole iron source, whereas *CFEM2*, *CFEM3* and to a lesser degree *CFEM6* are clearly required. The basal level of expression of the CFEM family in the absence if *BCR1* is therefore sufficient for survival of *C. parapsilosis* on hemin. In *C. albicans*, Bcr1 may be more important for iron utilization because a deletion grows slowly when heme is the only source of iron present (Figure 4A), although the reduction of growth is not as dramatic as when the *RBT5* is deleted [28]. The role of the CFEM family in iron acquisition is therefore conserved between *C. albicans* and *C. parapsilosis*, and is likely to be an ancestral feature of the *Candida* clade.

We used global transcriptional profiling to investigate the role of the CFEM family in the response to iron. Our analysis confirmed the large levels of induction of *CFEM2*, *CFEM3* and *CFEM6*, which lie among the 20 genes with the greatest increases in expression (Table 1, Table S3). We did not identify increases in *CFEM4*, detected by RT-PCR (Figure 4C). Further analysis of the microarray data indicates that iron-dependent expression in *C. parapsilosis* is similar to *C. albicans* [38], [39]. When iron is depleted, expression of components of the reductive transport system (e.g. *FRE9*, *FRE10*, and several other potential ferric reductases) is increased (Table 1). Expression of heme oxygenase is also increased, whereas expression of many respiratory protein genes is reduced. It is likely that at least some of the same transcription factors control the transcriptional response to iron in both species, because an ortholog of *SFU1* (a GATA-type repressor of transcription in *C. albicans* in high iron conditions [38] and of *HAP43* (a member of the CCAAT-binding complex, an iron-dependent repressor in *C. albicans* [43], [44], is reduced. Expression of *SEF1*, which was recently shown to be an activator of iron-uptake genes in *C. albicans* [39] is increased (Table 1, Table S3). The regulatory circuit described for Sfu1, Sef1 and Hap43 in *C. albicans* [39] is therefore also likely to function in *C. parapsilosis*. In *C. albicans*, expression of several of the CFEM genes is directly controlled by Sef1, and some are also regulated by Sfu1 [39]. It is highly likely that these factors are also required for the iron-dependent expression of *CFEM2*, *CFEM3*, *CFEM4* and *CFEM6* that we observed in *C. parapsilosis*.

Expression of *CPAG_02488*, the sole *C. parapsilosis* homolog of the *S. cerevisiae CTH1/CTH2* genes, is induced in low iron (Table 1). In *S. cerevisiae* these genes encode RNA binding proteins that control the degradation of mitochondrial-associated mRNAs in response to iron levels [45], [46], [47]. It is therefore likely that the iron-response in *Candida* species is also regulated by post-translational mechanisms, similar to *S. cerevisiae*.

In *C. albicans*, deleting *RBT5*, *PGA10* or *CSA1* does not have any effect on very early stage biofilms, but by 8 h a defect is obvious [25]. The biofilms generated were very fragile, and detached easily from the surface. Deleting all three genes resulted in more severe defects. The fragile biofilms generated resemble those produced by *bcr1* knockouts in *C. albicans* [17], [18]. Because Bcr1 regulates biofilms in both *C. albicans* and *C. parapsilosis* and also controls expression of CFEM genes in both species, we expected that the CFEM family in *C. parapsilosis* would play a role in biofilm growth in this species. We were therefore surprised that the three *CFEM* knockouts we generated in *C. parapsilosis* have no effect on biofilms (Figure 3). There are 7 members of the CFEM family in *C. parapsilosis*, and although we tested the major targets of Bcr1 (Figure 2C) it is possible that other family members are required for biofilm growth. However, there is clearly a difference in the behavior of the CFEM deletions in biofilm growth in the two species. We also find that the biofilm defects in the *BCR1* deletions in the two species are not identical. The *C. albicans* knockout generates biofilms that are very fragile and easily washed off the substrate, whereas there is little evidence for any biofilm formation at all in the *C. parapsilosis bcr1* knockout [16]. Yi et al [48] have recently demonstrated that in *C. albicans*, Bcr1-dependent biofilm formation is also affected by mating type. Whereas Bcr1 is required for biofilm formation by **a**/alpha cells, it does not play a role in biofilms generated by **a/a** cells. All the *C. parapsilosis* biofilms described here are generated by **a/a** cells, and it is very likely that this species has only *MTL* **a** idiomorphs [49], [50]. It is therefore likely that Bcr1 has a species-specific role in biofilm formation.

The ortholog of Bcr1 in *S. cerevisiae* is Usv1which regulates genes involved in non-fermentative growth and salt stress [51], and has been predicted to regulate genes important for protein folding during stationary phase [52]. This is substantially different to the role proposed in *C. albicans*, and here in *C. parapsilosis*. To further study changes in Bcr1 function within *Candida* species, it will be necessary to identify the binding sites in the promoters of target genes. Many Bcr1-regulated genes in *C. albicans* are controlled by several proteins. For example, expression of *RBT5* is repressed by Tup1, Hog1 and Sfu1, and induced by Rfg1 and Rim101 [38], [53], [54], [55], [56]. Identification of the direct target sites of Bcr1 in each species will therefore require direct analysis of bound genes.

Comparative genomic analysis is an important predictor of gene function. Comparisons between *S. cerevisiae* and *C. albicans* have been very helpful in the study of transcriptional regulation, such as dissecting the roles of Gat1, a regulator of nitrogen utilization in both species [57], and Upc2, which controls expression of ergosterol biosynthesis genes [58]. The role of Upc2 is also conserved in *C. parapsilosis* (Guida et al, submitted). However, conservation of sequence is not always indicative of conservation of function. Our analysis suggests that the role of the CFEM family in acquisition of iron from heme may be an ancient or ancestral function. However, their role in biofilm formation may be restricted to *C. albicans*, perhaps related to the formation of hyphae in this species. There is also increasing evidence that transcriptional rewiring is a major component of evolutionary change [59]. For example, in *C. albicans*, the transcription factor Cph1 is required for expression of the galactose pathway, replacing the role of Gal4 in *Saccharomyces* species [60]. The role of Mcm1 in regulation of mating and other genes differs substantially between *C. albicans* and *S. cerevisiae* [61], and the regulation of ribosomal protein expression is also significantly different [62], [63]. Whereas the *Saccharomyces* and *Candida* lineages are fairly distant relatives [64], even within closely related species there is considerable variation in transcription factor binding [65]. Our results suggest that there has been some rewiring of the Bcr1 regulon between the closely related species *C. albicans* and *C. parapsilosis*, and that only regulation of the CFEM family is conserved.

## Materials and Methods

### Ethics statement

All animal work was conducted with respect to the relevant guidelines in Ireland and the American Association for Accreditation of Laboratory Care criteria. Ethical approval was obtained from the Animal Research Ethics Subcommittee at University College Dublin (P-08-55), and the Animal Research Committee of the William S. Middleton Memorial Veterans Administration Hospital (MV1947-0-01-11).

### Strains and media

*C. parapsilosis* strains (Table S5) were routinely grown in YPD medium (1% yeast extract, 2% peptone, 2% dextrose) at 30°C. To determine the effect of reduced iron, a single colony was inoculated in 5 ml of YPD overnight, and then diluted 5-fold in YPD supplemented with 200 µg/ml of BPS. The culture was incubated at 200 rpm for 5 h at 30°C, then washed and resuspended in an equal volume of PBS. 5 µl was spotted on YPD agar and where noted supplemented with 1 mM BPS or 2 µM Hemin (Fluka). Biofilms were developed on silicone squares pre-treated with 10% FCS (fetal calf serum) in synthetic defined (SD) medium supplemented with 50 mM glucose at 37°C. For RNA extraction for the Bcr1 profiling experiment, cells were grown in SD medium supplemented with 50 mM glucose and 10% FCS at 37°C. For plate tests, overnight cultures were diluted 5-fold in YPD supplemented with 200 µM BPS, and incubated for 5 h at 30°C. Cells were then washed three times with PBS buffer, and resuspended in equal volume of PBS buffer. 5 µl of successive dilutions of each cell culture was spotted on the agar plates. Agar plates were incubated in the dark at 30°C for 14 days. All pictures were taken on the same day and at the same magnification.

### In vivo biofilm growth

Biofilms were developed *in vivo* using a rat central venous catheter model, as described previously [35]. Catheters were removed after 24 h. Sections were cut and examined using Scanning Electron Microscopy (SEM).

### Generation of gene knockouts

The sequences of oligonucleotide primers are listed in Table S5. The generation of *his1Δ/ura3Δ* and *bcr1Δ* strains are described in Figure S1.

*CFEM2* and *CFEM3* are adjacent genes in *C. parapsilosis* and were disrupted simultaneously using *URA3* and *HIS1*. Oligonucleotides Cp25/26UH_F and Cp25/26UH_R were used to amplify *URA3* and *HIS1*, and the purified PCR products were transformed into the CDUH3 strain by electroporation. *CFEM2* and *CFEM3* were then re-introduced separately into the double mutant using the *SAT1*-flipper cassette. To reintegrate *CFEM2*, a 3.3 kb fragment including the entire *CFEM2* ORF, 2.2 kb upstream and 404 downstream sequence was amplified using oligonucleotides Cp25/26_SacII and Cp25/26_SacI, and was then cloned into plasmid pCD8 to generate pCD47. The upstream sequence from *CFEM3* was amplified using oligonucleotides Cp25/26_KpnI and Cp25/26_ApaIRE, and the fragment was cloned into plasmid pCD47 to generate pCD48. To reintegrate *CFEM3*, the entire *CFEM3* open reading frame (ORF) including 449 bp of upstream sequence and 378 bp of downstream sequence was amplified using oligonucleotides Cp25/26_KpnI and Cp25/26_ApaI, introducing restriction sites *Kpn*I and *Apa*I, respectively. The fragment was then cloned into plasmid pCD8 downstream from the *SAT1* cassette to generate pCD44. A 597 bp fragment downstream from *CFEM2* was amplified using oligonucleotides Cp25/26_SacIIRE and Cp25/26_SacI, and cloned between restriction sites *Sac*I and *Sac*II in pCD44 to generate pCD45. Both plasmids pCD45 and pCD48 were linearized using restriction enzymes *Pvu*I and *Sac*I, and the fragments were transformed by electroporation. Strains harboring the correct integrations were then manipulated to recycle the *SAT1* cassette as described previously by Ding and Butler [16].

*CFEM6* was also disrupted using *URA3* and *HIS1*. Oligonucleotides 2874UHF and 2874UHR were used to amplify *URA3* or *HIS1* from plasmid pLUL2 and pLHL2, respectively. The PCR products were purified and transformed into *C. parapsilosis* CDUH3 by electroporation. We also knocked out *CFEM6* using the *SAT1*-flipper cassette. A 468 bp fragment, including 236 bp of upstream sequence and 232 bp coding sequence from *CFEM6*, was amplified using oligonucleotides 73/74Kpn2 and 73/74Apa, which introduce restriction sites *Kpn*I and *Apa*I. A 501 bp fragment (3′ to *CFEM6*) was amplified using oligonucleotides 73/74SII and 73/74SI, which introduce restriction sites *Sac*II and *Sac*I. Both fragments were cloned between *Kpn*I and *Apa*I sites, and *Sac*II and *Sac*I sites respectively to generate plasmid pCD54. The *Kpn*I/*Sac*I fragment was gel purified and transformed into the wildtype strain (*C. parapsilosis* CLIB214) to knock out the first allele of *CFEM6*. To increase the efficiency of disruption of the second allele, a different region was deleted using plasmid pCD55. A 486 bp fragment, including 155 bp coding sequence and 331 bp downstream of *CFEM6* was amplified using oligonucleotides 74NSII and 74NSI, introducing restriction sites *Sac*II and *Sac*I. This was then cloned between *Sac*II and *Sac*I sites on pCD54 substituting the original fragment, to generate the plasmid pCD55. The second allele was then deleted as above.

Southern blots were carried out using DIG High Prime DNA Labeling and Detection Starter Kit II (Roche). 20 µg of genomic DNA from the wildtype and from the *CFEM2/3* knockouts (CLIB214, CDUH2526his, CDUH25/26, CD26, CD25, CD262, and CD254) were digested with *Eco*RI/*Eco*RV. For *CFEM2/CFEM3*, a probe was amplified using Cp25/26_KpnI and Cp25/26_ApaIRE, which binds to a region of *CFEM3* upstream from the integration site. This detects a 4.18-kb fragment from the wildtype allele, and a 3.5 kb fragment from replacement by *URA3* or *HIS1*. Substitution of *HIS1* with *CFEM3* and the *SAT1* cassette generates a 4.3 kb fragment, which is reduced to 4.18 kb when the cassette is removed. Substitution with *CFEM2* and the *SAT1* cassette generates a 3.27 kb fragment, which is reduced very slightly (3.25 kb) when the cassette is removed.

To confirm the *CFEM6* knockouts, genomic DNA was digested with *Sac*I. A probe was amplified from the 3′ end of *CFEM6* using oligonucleotides 2874probeF and 2874probeR. This detects a 4.2-kb fragment from the wildtype allele of *CFEM6*. A 2.9-kb fragment is generated when *URA3* and *HIS1* are integrated at *CFEM6*. The integration of either *SAT1*-flipper cassette at *CFEM6* results a 5.9-kb fragment. Recycling of the pCD54 construct results in a 1.9-kb fragment, and recycling of pCD55 integration results a 1.7-kb fragment.

### DNA microarrays and RT-PCR

Cells were grown for 5 hours in SD medium supplemented with 50 mM glucose and 10% FCS at 37°C. RNA samples used in DNA microarrays and RT-PCR were extracted using a Ribopure kit (Ambion). The DNA microarrays were manufactured by Agilent Technologies and represent 5,834 ORFs for *C. parapsilosis* and 6,101 ORFs for *C. albicans* [36], [66]. cDNA synthesis and labeling were carried out as described previously [36]. Seven biological replicates were used for *C. parapsilosis*; in five the *BCR1* knockout generated using *SAT1*-flipper cassette samples (Cdb71) were labeled with Cy5, and in two the knockout generated using *URA3/HIS1* (CDUHB6) were labeled with Cy3. Both knockouts were compared to the same wildtype (CLIB214) labeled with Cy3 or Cy5 where appropriate. Four biological replicates comparing *C. albicans bcr1Δ* and wild-type strains (CJN702 and DAY286, from A. Mitchell) were also examined by microarray, two of which were dye swaps.

cDNA hybridization, washing and scanning procedures were carried out as described previously [36]. To determine the transcriptional response of *C. parapsilosis* to low iron, overnight cultures in YPD were diluted to an *A_600_* of 0.2 in 100 ml SD medium and the culture was incubated at 37°C for 5 h before RNA extraction. In five replicates, two BPS treated samples were labeled with Cy5, and three were labeled with Cy3 (dye swaps). Two samples without BPS treatment were labeled with Cy3, and three were dye swaps. Quantitative RT-PCR assays were carried out as described previously [36].

### Data analysis

Profiling experiments were carried out using *C. albicans* or *C. parapsilosis* arrays manufactured with tools available from Agilent eArray [36]. This microarray platform is described in the NCBI Gene expression Omnibus Database (GEO) (GPL7693). Each ORF is represented by two probes, both spotted in duplicate.

Data was analyzed was using the LIMMA package [67] from the Bioconductor Project (http://bioconductor.org). The datasets were preprocessed by applying Lowess normalization and no background correction (as suggested in [68]). The duplicated probes within each array were considered as technical replicates. This assumption allows us to take full advantage of the platform design, analyzing the within-array replicate spots using a pooled correlation method. For the *C. albicans* and *C. parapsilosis bcr1Δ* versus wildtype experiments, the final lists of differentially expressed genes were generated by selecting probes with an adjusted p-value less than 0.01, and a fold-change greater than 2 (Table S1 and Table S2). For the iron depletion study, the final list of 149 genes was generated by selecting probes with a fold-change greater than 1.5 and p-value lower than 0.05 (Table S3).

*C. albicans* orthologs were extracted from the Candida Gene Order Browser [69], Maguire et al, in preparation. Raw microarray data and the description of the array have been deposited in the Gene Expression Omnibus database under the accession number GSE33490, according to the MIAME guidelines.

5,214 *C. parapsilosis* orthologs of *C. albicans* genes were identified (84.2% of the *C. albicans* genome). 83 of 149 genes differentially expressed in the iron-depletion arrays have an ortholog in *C. albicans*. All the GO term enrichment analysis were performed using the web application “GO term finder” available on the “Candida Genome Database” (CGD, http://www.candidagenome.org). The background for the test was appropriately adjusted by excluding those *C. albicans* genes with no *C. parapsilosis* ortholog.

## Supporting Information

Figure S1 **Generation of** ***bcr1*** **deletion in** ***C. parapsilosis*** **.**
(DOC)

Table S1 ***C. parapsilosis*** **genes with differential expression in** ***bcr1*** **deletion grown in SD +FBS.**
(XLS)

Table S2 ***C. albicans*** **genes with differential expression in** ***bcr1*** **deletion grown in SD +FBS.**
(XLS)

Table S3 ***C. parapsilosis*** **genes with differential expression in low iron at 37 degrees.**
(XLS)

Table S4 **Genes in intersection of expression profiles in** **Figure 2A** **.**
(XLS)

Table S5 **List of strains and oligonucleotide primers.**
(DOC)
