# Supplementary material for: Conserved and Divergent Roles of Bcr1 and CFEM Proteins in Candida parapsilosis and Candida albicans
Source: PLoS One. 2011 Dec 1;6(12):e28151. doi: 10.1371/journal.pone.0028151 (PMC3228736; doi:10.1371/journal.pone.0028151)
Supplement: Figure S1 — Generation of bcr1 deletion in C. parapsilosis . (DOC) [file pone.0028151.s001.doc]

**Supplementary Material**

We previously described the construction of a *ura3* deletion in *C. parapsilosis* and here we used a similar approach to generate a *his1Δ/ura3Δ* strain. *HIS1* was disrupted using the *SAT1*-flipper cassette . Due to the low efficiency of integration at *HIS1*, the two alleles were disrupted using different constructs (pCD35 and pCD37). For plasmid pCD35, a 405-bp fragment from the upstream region of *HIS1* (including 354 bp of promoter sequence and 51 bp coding sequence) was amplified using oligonucleotides BUT316 and BUT317, introducing restriction sites *Kpn*I and *Apa*I respectively, which was then cloned into plasmid pCD8 , generating pCD33. A 423-bp fragment from the downstream region of *HIS1* was amplified using oligonucleotides BUT318 and BUT319, and was cloned between restriction sites *Sac*II and *Sac*I on plasmid pCD33 to generate pCD35. The *SAT1* cassette was isolated from plasmid pCD35 by digestion with *Kpn*I and *Sac*I, and the fragment was introduced into *ura3* deletion strain CDU1 to delete the first *HIS1* allele. The *SAT1* cassette was then recycled from CDUhis1, generating the heterozygote knockout CDUhis11. For plasmid pCD37, a 439-bp fragment from the upstream region of *HIS1* (including 110 bp of promoter sequence and 329 bp of coding sequence) was amplified from *C. parapsilosis* genomic DNA using oligonucleotides HisKpn2 and HisApa2, and the fragment was cloned between restriction sites *Kpn*I and *Apa*I in plasmid pCD35 to generate plasmid pCD37. The cassette was released from pCD37 by digestion with *Kpn*I and *Sac*I, and the purified fragment was introduced into CDUhis11 to generate strain CDUH1. Recycling of the cassette from CDUH1 generated CDUH3.

**Verification of gene disruption by PCR and Southern blot analysis**

Southern blots were used to confirm the *his1* deletion. 20 μg of genomic DNA from the wildtype and *HIS1* knockout strains (CLIB214, CDUhis1, CDUhis11, CDUH1, and CDUH3) were digested with *Hinc*II. A probe was amplified from *C. parapsilosis* genomic DNA using BUT318 and BUT319, which binds to a region of *HIS1* downstream from the integration site. Labeling and hybridizations were carried out using a DIG High Prime DNA Labeling and Detection Starter Kit II (Roche).

The *BCR1* knockout generated using *URA3* and *HIS1* was confirmed by PCR. Oligonucleotides BUT261 binds upstream from *BCR1* and URAR binds within the *URA3* cassette, generating a fragment of 1.25 kb. Oligonucleotides BUT261 and HISR, which bind within *HIS1*, generate a fragment of 1.25 kb. Oligonucleotide BUT261 and BUT262 (from within the *BCR1* ORF) were used to confirm the deletion of both alleles.


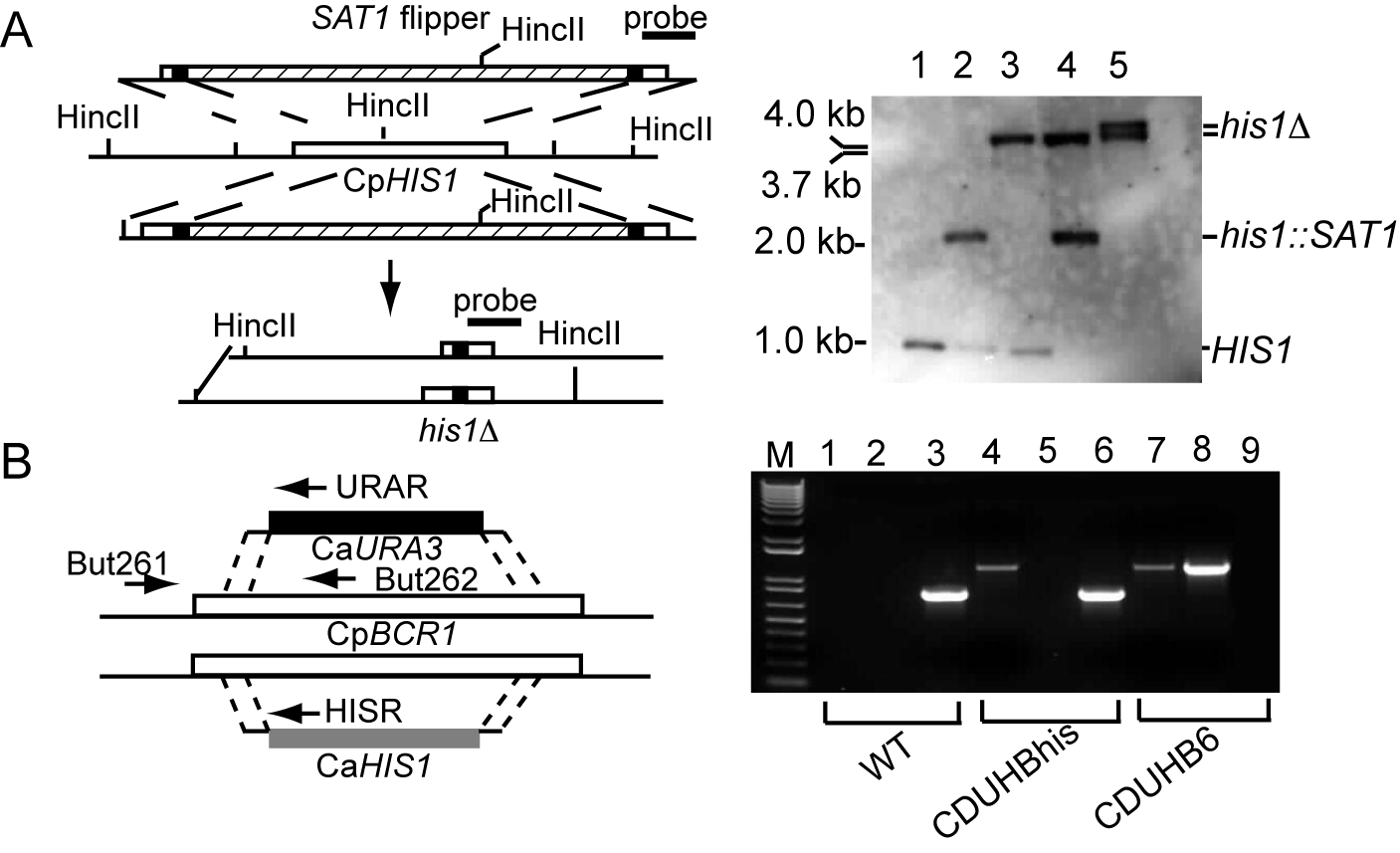


Figure S1. Deleting *HIS1* and *BCR1* in *C. parapsilosis*.

1. *HIS1* was knocked out in a *ura3* null strain of *C. parapsilosis* (CDU1 ) using the *SAT1-*flipper cassette, to generate a *ura3*Δ*his1*Δ strain (CDUH3).Two different constructs were used to disrupt the two *HIS1* alleles, as described in Methods. Genomic DNA was isolated from five strains, digested with *Hinc*II and hybridized with a probe from the downstream region from *HIS1*. The wildtype allele generates a1.0 kb fragment. Integration of the cassettes at *HIS1* produces a 2.0 kb fragment. Recycling of the first cassette generates a 3.7 kb fragment, and recycling of the second cassette results a 4.0 kb fragment. Lane 1: *C. parapsilosis* CLIB214; Lane 2: *C. parapsilosis* CDUhis1 (*his1Δ::SAT1-FLP/ HIS1*); Lane 3: *C. parapsilosis* CDUhis11 (*his1Δ::FRT/ HIS1*); Lane 4: *C. parapsilosis* CDUH1 (*his1Δ::FRT/ his1Δ::SAT1-FLP*); and Lane 5: *C. parapsilosis* CDUH3 (*his1Δ::FRT/ his1Δ::FRT*). All strains (apart from CLIB214) are also carrying *ura3* deletions.
2. *BCR1* was disrupted by replacement with *URA3* and *HIS1* genes from *C. albicans*, amplified by PCR and transformed into *C. parapsilosis* CDUH3 by electroporation. Integration of the *HIS3* gene was confirmed by PCR using oligonuleotides But261 and HISR that generates a 1.26 kb fragment which is absent in the wild-type strain. Integration of *URA3* was confirmed using oligonuleotides But261 and URAR, which generates a 1.25 kb fragment. The deletion of *BCR1* was confirmed using oligonucleotides But261 and But262, which generate a 0.8 kb fragment from the wildtype allele. Lanes 1,4,7: But261/HISR; Lanes 2,5,8: But261/URAR; Lanes 3,6, 9: But261/But262. WT = CDUH3, CDUHBhis (*bcrΔ1::HIS1/BCR1*), CDUHB6 (*bcrΔ1::HIS1/ bcrΔ1::URA3*).

Ding, C. & G. Butler, (2007) Development of a gene knockout system i*n Candida parapsilos*is reveals a conserved role fo*r BC*R1 in biofilm formation*. Eukaryot Ce*l**l** 6: 1310-1319.
