# Supplementary material for: Conserved and Divergent Roles of Bcr1 and CFEM Proteins in Candida parapsilosis and Candida albicans
Source: PLoS One. 2011 Dec 1;6(12):e28151. doi: 10.1371/journal.pone.0028151 (PMC3228736; doi:10.1371/journal.pone.0028151)
Supplement: Table S5 — List of strains and oligonucleotide primers. (DOC) [file pone.0028151.s006.doc]

Table S5. List of strains and oligonucleotide primers.

| Oligo name | Applications | Target | Sequence (5’ to 3’) |
| --- | --- | --- | --- |
| BUT316 | *SAT1-FLP* knockout*SAT1*-*FLP* knockou | CpHIS1 | GGGGGGTACCGCGACACTGACTTGGAGTTG |
| BUT317 | *SAT1-FLP* knockout | CpHIS1 | GGGGGGGCCCTTTTGGGACTGCAAACAACA |
| HisKpn_2 | *SAT1-FLP* knockout | CpHIS1 | GGGGGGTACCCGTGTCCTCACTGAGTCATCA |
| HisApa_2 | *SAT1-FLP* knockout | CpHIS1 | GGGGGGGCCCTTTTTCTGGTACTTGTACTTGAAGTTT |
| BUT318 | *SAT1-FLP* knockout | CpHIS1 | GGGGCCGCGGCCATTATGGCTGAGTGTTTGAA |
| BUT319 | *SAT1-FLP* knockout | CpHIS1 | GCCATATGGAGCTCATGGATAT |
| BUT320 | Screening | CpHIS1 | GAAAAGTTGTGGCCCGAGTTCAAA |
| BUT321 | Screening | CpHIS1 | ACCAATTAGATCTTTCGGTTGG |
| CpBCR1UH_F | *URA3/HIS1* knockout | CpBCR1 | AGGAATAAATTCATTACCACCAATATCTGCCCACCACTACTCGTCTCATCGTCCTTTTGGTAGTTCCCTGCAACTGCCACCACAAT GGTAACGCCAGGGTTTTCCC |
| CpBCR1UH_R | *URA3/HIS1* knockout | CpBCR1 | TTGGACGAATGGAAGTTGCCTGGTATAGGCAAATTTTTAATTGAGGGTAAACTATTATTATTATTGAGATCATGTGTTTGGGTTGTAAACAGCTATGACCATGATT |
| Cp25/26UH_F | *URA3/HIS1* knockout | CFEM2+3 | ACTGATAACAGCGAGAGTGTTTCAGAGACTAGTGATAGCAGTAGTGCATCAGCTACTGATTCTGATGAAGAGACTTCAGCGGTAACGCCAGGGTTTTCCC |
| Cp25/26UH_R | *URA3/HIS1* knockout | CFEM2+3 | GGGCGGATGATGAAGATTGTTCTTCAGCACTTGATGGCTTTTCTTCTTGAGAAGATTCAGCAGGAGCAGAAGATTCAGCAAAACAGCTATGACCATGATT |
| 2874UHF | *URA3/HIS1* knockout | CFEM6 | CCTCCCACCATTGGACTCTTAAAAAAACGATATATTTGAAATACATCACACACACACAACCACCTCAATCGCTTAGCAAGGGTAACGCCAGGGTTTTCCC |
| 2874UHR | *URA3/HIS1* knockout | CFEM6 | CGAGGCTCTCAAACATCTCATTTGTACAAAGCTTCGATTTTGGACCGAAAGTGAAAAGTTGCACAAAACAATCCGAATCA AAACAGCTATGACCATGATT |
| 73/74Kpn2 | *SAT1-FLP* knockout | CFEM6 | GGGGGGTACCGCAAAACTGCATCTGATTGG |
| 73/74Apa | *SAT1-FLP* knockout | CFEM6 | GGGGGGGCCCAGTACGGACATGGCGTAACA |
| 74SI | *SAT1-FLP* knockout | CFEM6 | GGGGGAGCTCAAATCGTTCTATGTACTTTTGCCATA |
| 74SII | *SAT1-FLP* knockout | CFEM6 | GGGGCCGCGGTTCGGATTGTTTTGTGCAAC |
| 74NSI | *SAT1-FLP* knockout | CFEM6 | GGGGGAGCTCCGCTCTTGAATTCTCCTCCA |
| 74NSII | *SAT1-FLP* knockout | CFEM6 | GGGGCCGCGGAAAGTGAGGCTGCAAAAGCTCA |
| Cp25/26_Check | Screening | CFEM2+3 | GTTGATGACTGAAAGTCAGTGTTG |
| Cp25/26_KpnI | *SAT1-FLP* reintegration | CFEM2+3 | GGGGGGTACCTAGGCACACCACACACTTTTTG |
| Cp25/26_ApaI | *SAT1-FLP* reintegration | CFEM2+3 | GGGGGGGCCCTCTTTTTAATGGAACCACTCCTG |
| Cp25/26_SacII | *SAT1-FLP* reintegration | CFEM2+3 | GGGGCCGCGGAAGGTACCCTTTAGGTCTAGGT |
| Cp25/26_SacI | *SAT1-FLP* reintegration | CFEM2+3 | GGGGGAGCTCTTCATCAATGCAATCCAATGT |
| Cp25/26_ApaRE | *SAT1-FLP* reintegration | CFEM2+3 | GGGGGGGCCCGGCGGCTTGTACAAATGATA |
| Cp25/26_SacIIRE | *SAT1-FLP* reintegration | CFEM2+3 | GGGGCCGCGGTGAATCTTCTGCTCCTGCTG |
| Cp25/26_SacIRE | *SAT1-FLP* reintegration | CFEM2+3 | GGGGGAGCTCTTGGTATTCGCCTTTGTTCC |
| URAR | Screening | CaURA3 | TATAAACCAGTGTGTATGGGGTTG |
| HISR | Screening | CaHIS1 | TCGGTAGTTGGTGGTTAAGTAAAAG |
| ACT1 for | qRT-PCR | CpACT1 | GTGACGAAGCCCAATCAAAGA |
| ACT1 rev | qRT-PCR | CpACT1 | CATATCATCCCAGTTGGAAACGA |
| cpar1324 F | qRT-PCR | CFEM1 | CCGGCTCTACTCCATGTCCAT |
| cpar1324 R | qRT-PCR | CFEM1 | TTGACCAATAGCACCACCAAATT |
| cpar1325 F | qRT-PCR | CFEM2 | TGGTTCAACCCCATGTCCATA |
| cpar1325 R | qRT-PCR | CFEM2 | TACCAATGGCACCAGCAAATT |
| cpar1326 F | qRT-PCR | CFEM3 | AAAGACGGCAATGCAATGG |
| cpar1326 R | qRT-PCR | CFEM3 | TCGCTGTTATCAGTTTCCGAAAC |
| cpar1327 F | qRT-PCR | CFEM4 | ACTGGCAACACGCCTTGTC |
| cpar1327 R | qRT-PCR | CFEM4 | TAAACCTGACCATTGTGGCATAA |
| cpar2873 NF | qRT-PCR | CFEM5 | TGTAACCAGTGGTGGTGAATCtgtaaccagtggtggtgaatc tgtaaccagtggtggtgaatcT |
| cpar2873 NR | qRT-PCR | CFEM5 | TCAGCTGATTGCGTTGTTTC |
| cpar2874 F | qRT-PCR | CFEM6 | TCTGCTGAAGAGACATCCTCTG |
| cpar2874 R | qRT-PCR | CFEM6 | GAAGACGAAGATGGAGCTTGAC |
| cpar2920 F | qRT-PCR | CFEM7 | TCAACACCTTGCCCATACTG |
| cpar2920 R | qRT-PCR | CFEM7 | AGCCAATGAGGTTGCTGAAC |
| CaACT1 for | qRT-PCR | CaACT1 | TTGGTGATGAAGCCCAATCC |
| CaACT1 rev | qRT-PCR | CaACT1 | CATATCGTCCCAGTTGGAAACA |
| orf9.5635 for | qRT-PCR | CaPGA7 | CACCCCTTGTCCATATTGGG |
| orf9.5635 rev | qRT-PCR | CaPGA7 | CATTGTGGCATCACGCAGA |
| orf9.5636 for | qRT-PCR | CaRBT5 | CATCAACGGTTTCGCTGACA |
| orf9.5636 rev | qRT-PCR | CaRBT5 | TTGGCACATTCTGGCAATTG |
| orf9.7114 for | qRT-PCR | CaCSA1 | TCGTTGGCTGCTGTACAATC |
| orf9.7114 rev | qRT-PCR | CaCSA1 | CTTCAGCAACAGCAGTTTGG |
| 2874probeF | Southern | CFEM6 | TGATCATTTCGAGTTGTTTTTG |
| 2874probeR | Southern | CFEM6 | GTCTCGCCTAACTCGCTTGT |

| Strains | Genotype | Description |
| --- | --- | --- |
| *C. parapsilosis* |  |  |
| CLIB214 | Wildtype | Type strain |
| CDU1 | *ura3Δ::FRT/ ura3Δ::FRT* | Ding and Butler, 2007 |
| CDUhis1 | *ura3Δ::FRT/ ura3Δ::FRT, his1Δ::SAT1-FLP/ HIS1* | derived from CDU1 |
| CDUhis11 | *ura3Δ::FRT/ ura3Δ::FRT, his1Δ::FRT/ HIS1* | derived from CDUhis1 |
| CDUH1 | *ura3Δ::FRT/ ura3Δ::FRT, his1Δ::FRT/ his1Δ::SAT1-FLP* | derived from CDUhis11 |
| CDUH3 | *ura3Δ::FRT/ ura3Δ::FRT, his1Δ::FRT/ his1Δ::FRT* | derived from CDUH1 |
| CDH4 | *ura3Δ::FRT/ URA3, his1Δ::FRT/ his1Δ::FRT* | derived from CDU1 |
| CDUHBhis | *ura3Δ::FRT/ ura3Δ::FRT, his1Δ::FRT/ his1Δ::FRT bcr1Δ::HIS1/BCR1* | derived from CDUH3 |
| CDUHB6 | *ura3Δ::FRT/ ura3Δ::FRT, his1Δ::FRT/ his1Δ::FRT*  *bcr1Δ::HIS1/ bcr1Δ::URA3* | derived from CDUHBhis |
| CDUH2526his | *ura3Δ::FRT/ ura3Δ::FRT, his1Δ::FRT/ his1Δ::FRT,*  *cfem2+3Δ::HIS1/ CFEM2+3* | derived from CDUH3 |
| CDUH25/26 | *ura3Δ::FRT/ ura3Δ::FRT, his1Δ::FRT/ his1Δ::FRT,*  *cfem2+3Δ::HIS1/ cfem2+3Δ::URA3* | derived from CDUH2526his |
| CD26 | *ura3Δ::FRT/ ura3Δ::FRT, his1Δ::FRT/ his1Δ::FRT,*  *cfem2Δ+CFEM3::SAT1-FLP / cfem2+3Δ::URA3* | derived from CDUH25/26 |
| CD262 | *ura3Δ::FRT/ ura3Δ::FRT, his1Δ::FRT/ his1Δ::FRT,*  *cfem2Δ+cfem3::FRT / cfem2+3Δ::URA3* | derived from CD26 |
| CD25 | *ura3Δ::FRT/ ura3Δ::FRT, his1Δ::FRT/ his1Δ::FRT,*  *cfem2+cfem3Δ::SAT1-FLP / cfem2+3Δ::URA3* | derived from CDUH25/26 |
| CD254 | *ura3Δ::FRT/ ura3Δ::FRT, his1Δ::FRT/ his1Δ::FRT,*  *cfem2+cfem3Δ::FRT / cfem2+3Δ::URA3* | derived from CD25 |
| CD74U2 | *ura3Δ::FRT/ ura3Δ::FRT, his1Δ::FRT/ his1Δ::FRT, CFEM6/cfem6Δ::URA3* | derived from CDUH3 |
| CD74UH1 | *ura3Δ::FRT/ ura3Δ::FRT, his1Δ::FRT/ his1Δ::FRT,*  *cfem6Δ::HIS1/cfem6Δ::URA3* | derived from CD74U2 |
| CD741 | *cfem6Δ::SAT1-FLP/CFEM6* | derived from CLIB214 |
| CD745 | *cfem6Δ::FRT/CFEM6* | derived from CD741 |
| CD746 | *cfem6Δ::FRT/cfem6Δ::SAT1-FLP* | derived from CD745 |
| CD749 | *cfem6Δ::FRT/cfem6Δ::FRT* | derived from CD746 |
| *C. albicans* |  |  |
| SC5314 | Wildtype | Nobile et al, 2006 |
| CJN702 | *ura3::imm434/ura3::imm434, arg4::hisG/arg4::hisG, his1::hisG/his1::hisG::pHIS1, bcr1::ARG4/bcr1::URA3* | Nobile et al, 2006 |
